# Update to “Homologous Recombination Repair Defect May Predict Treatment Response to Peptide Receptor Radionuclide Therapy for Neuroendocrine Tumors”

**DOI:** 10.1093/oncolo/oyac068

**Published:** 2022-04-11

**Authors:** Mojun Zhu, Tanios Bekaii-Saab

**Affiliations:** Mayo Clinic, Rochester, MN, USA; Mayo Clinic, Phoenix, AZ, USA

## Abstract

This letter to the editor reports subsequent rechallenge with peptide receptor radionuclide therapy (PRRT) in a patient with pancreatic neuroendocrine tumor bearing BRCA1 mutation previously treated with PRRT.

Last year, we reported successful treatment of a grade 3 pancreatic neuroendocrine tumor (PNET; Ki-67 index 40%) with ^177^Lu-dotatate (4 treatments) in a patient with a pathogenic, heterozygous *BRCA1* germline mutation (c.68_69delAG).^1^ This patient initially received capecitabine and temozolomide (CAPTEM) with at best mixed response. Given the presence of *BRCA1* mutation, ^177^Lu-dotatate was administered as the next line of therapy, leading to resolution of bone pain and significant radiographic response in primary tumor and metastases in liver and bones that remained stable until new lesions revealed in liver and bones by a ^68^Ga-dotatate PET/CT scan 15 months later. Patient restarted CAPTEM but had radiographic disease progression after 2 cycles of treatment. Rechallenge with 2 treatments of ^177^Lu-dotatate were attempted 19 months after completion of the first course of peptide receptor radionuclide therapy (PRRT), and this again resulted in substantial improvement in the number and degree of uptake in the metastatic lesions with continued response on ^68^Ga-dotatate PET/CT 5 months after completion of therapy (Figure 1). Patient tolerated PRRT rechallenge well without grades 3-4 toxicities based on Common Terminology Criteria for Adverse Events version 5.0.

**Figure 1.**
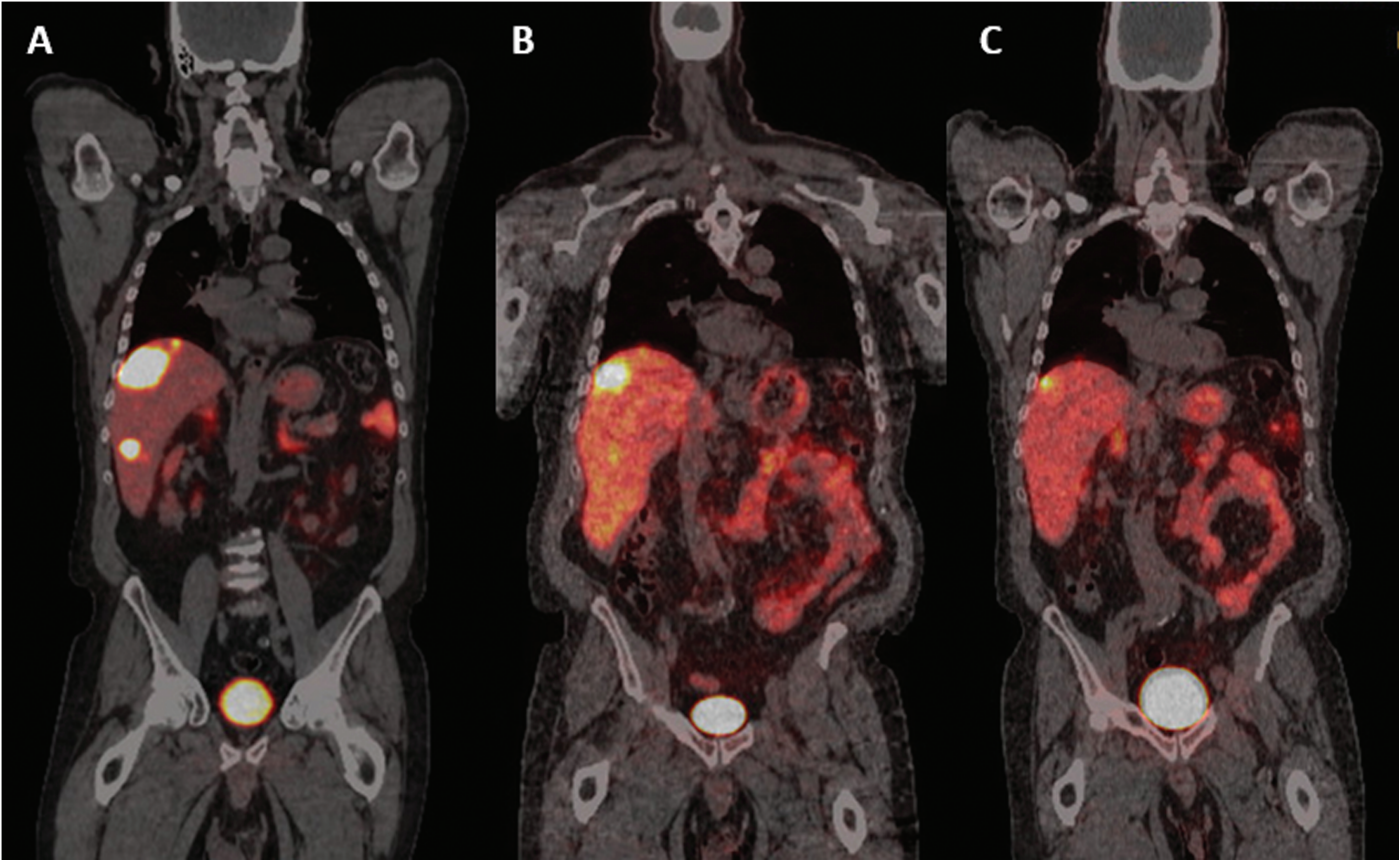
^68^Ga-Dotatate PET/CT of the patient: (**A**) before, (**B**) 1 month after, and (**C**) 5 months after PRRT rechallenge.

This case report supports that rechallenging NET patients with PRRT can be considered in those with at least stable disease to the first course of PRRT which usually consists of 4 treatments.^2^ In patients who were naïve to PRRT, grades 3-4 cytopenias occurred to about 10% of them during treatment^3^ and the incidence of PRRT-induced myeloid neoplasms was estimated to be 2.6% with most diagnoses made 1 year after treatment.^4^ The safety and efficacy of PRRT rechallenge have not been examined prospectively, but hematological toxicities did not appear increased based on retrospective studies^4-13^ and a disease control rate of about 70% was proposed by 2 meta-analyses despite significant between-study heterogeneity.^14,15^ PRRT rechallenge is therefore feasible and represents a reasonable option in patients who have no alternative therapies that meaningfully prolong survival.

In our opinion, patient selection for PRRT remains a challenge in clinical practice. Similar to other agents such as poly ADP-ribose polymerase (PARP) inhibitors that target tumor cell DNA in patients with defective homologous recombination repair (HRR), PRRT may impact not only the therapeutic efficacy of subsequent anti-cancer therapies but also accumulative, treatment-related toxicities which can lead to irreversible hematological malignancies. Thus, clinical trials that evaluate the potential of HRR as a predictive biomarker for PRRT and examine the long-term toxicity of DNA-damaging agents in patients with HRR are prudent.

## References

[1] Zhu M, Bassam Sonbol M, Halfdanarson T, et al. Homologous recombination repair defect may predict treatment response to peptide receptor radionuclide therapy for neuroendocrine tumors. Oncologist. 2020;25(8):e1246-e1248. 32510802 10.1634/theoncologist.2020-0029 PMC7418337

[2] Ambrosini V, Kunikowska J, Baudin E, et al. Consensus on molecular imaging and theranostics in neuroendocrine neoplasms. Eur J Cancer (Oxford, England: 1990). 2021;146:56-73. 10.1016/j.ejca.2021.01.008 PMC8903070 33588146

[3] Halfdanarson TR, Strosberg JR, Tang L, et al. The North American Neuroendocrine Tumor Society consensus guidelines for surveillance and medical management of pancreatic neuroendocrine tumors. Pancreas. 2020;49(7):863-881. https://doi.org/10.1097/MPA.0000000000001597. 32675783 10.1097/MPA.0000000000001597

[4] Sonbol MB, Halfdanarson TR, Hilal T. Assessment of therapy-related myeloid neoplasms in patients with neuroendocrine tumors after peptide receptor radionuclide therapy: A systematic review. JAMA Oncol. 2020;6(7):1086-1092. https://doi.org/10.1001/jamaoncol.2020.0078. 32297906 10.1001/jamaoncol.2020.0078

[5] Lim LE, Chan DL, Thomas D, et al. Australian experience of peptide receptor radionuclide therapy in lung neuroendocrine tumours. Oncotarget. 2020;11(27):2636-2646. 32676165 10.18632/oncotarget.27659 PMC7343632

[6] Rudisile S, Gosewisch A, Wenter V, et al. Salvage prrt with (177)lu-dota-octreotate in extensively pretreated patients with metastatic neuroendocrine tumor (net): dosimetry, toxicity, efficacy, and survival. BMC Cancer. 2019;19(1):788. 31395036 10.1186/s12885-019-6000-y PMC6686531

[7] Vida Navas EM, Martínez Lorca A, Sancho Gutiérrez A, et al. Case report: re-treatment with lu-dotatate in neuroendocrine tumors. Front Endocrinol. 2021;12:676973. 10.3389/fendo.2021.676973 PMC8082310 33935979

[8] van Essen M, Krenning EP, Kam BL, et al. Salvage therapy with (177)lu-octreotate in patients with bronchial and gastroenteropancreatic neuroendocrine tumors. J Nucl Med. 2010;51(3):383-390. 20150247 10.2967/jnumed.109.068957

[9] Sabet A, Haslerud T, Pape UF, et al. Outcome and toxicity of salvage therapy with 177lu-octreotate in patients with metastatic gastroenteropancreatic neuroendocrine tumours. Eur J Nucl Med Mol Imaging. 2014;41(2):205-210. 24030668 10.1007/s00259-013-2547-z

[10] Severi S, Sansovini M, Ianniello A, et al. Feasibility and utility of re-treatment with (177)lu-dotatate in gep-nens relapsed after treatment with (90)y-dotatoc. Eur J Nucl Med Mol Imaging. 2015;42(13):1955-1963. 26112388 10.1007/s00259-015-3105-7

[11] Yordanova A, Mayer K, Brossart P, et al. Safety of multiple repeated cycles of (177)lu-octreotate in patients with recurrent neuroendocrine tumour. Eur J Nucl Med Mol Imaging. 2017;44(7):1207-1214. 28246882 10.1007/s00259-017-3652-1

[12] Vaughan E, Machta J, Walker M, et al. Retreatment with peptide receptor radionuclide therapy in patients with progressing neuroendocrine tumours: efficacy and prognostic factors for response. Br J Radiol. 2018;91(1091):20180041. 29513039 10.1259/bjr.20180041 PMC6475926

[13] van der Zwan WA, Brabander T, Kam BLR, et al. Salvage peptide receptor radionuclide therapy with [(177)lu-dota,tyr(3)]octreotate in patients with bronchial and gastroenteropancreatic neuroendocrine tumours. Eur J Nucl Med Mol Imaging. 2019;46(3):704-717. 30267116 10.1007/s00259-018-4158-1 PMC6351514

[14] Kim YI. Salvage peptide receptor radionuclide therapy in patients with progressive neuroendocrine tumors: A systematic review and meta-analysis. Nucl Med Commun. 2021;42(4):451-458. 33346603 10.1097/MNM.0000000000001350

[15] Strosberg J, Leeuwenkamp O, Siddiqui MK. Peptide receptor radiotherapy re-treatment in patients with progressive neuroendocrine tumors: a systematic review and meta-analysis. Cancer Treat Rev. 2021;93:102141. 33418096 10.1016/j.ctrv.2020.102141

